# A leadless pacemaker in the real‐world setting: Patient profile and performance over time

**DOI:** 10.1002/joa3.12811

**Published:** 2023-01-08

**Authors:** Paul R. Roberts, Nicolas Clémenty, Pierre Mondoly, Stefan Winter, Pierre Bordachar, David Sharman, Werner Jung, Romain Eschalier, Cathrin Theis, Pascal Defaye, Christopher Anderson, Aimée Pol, Kiah Butler, Christophe Garweg

**Affiliations:** ^1^ University Hospital Southampton NHS Foundation Trust Southampton UK; ^2^ Centre Hospitalier Régional Universitaire de Tours Tours France; ^3^ Centre Hospitalier Universitaire de Toulouse Toulouse France; ^4^ St. Vinzenz Hospital Köln Germany; ^5^ Centre Hospitalier Universitaire de Bordeaux Bordeaux France; ^6^ Northampton General Hospital Cliftonville UK; ^7^ Schwarzwald‐Baar Klinikum Villingen‐Schwenningen Villingen‐Schwenningen Germany; ^8^ Université Clermont Auvergne and Cardiology Department, CHU Clermont‐Ferrand, CNRS SIGMA Clermont, Institut Pascal Clermont‐Ferrand France; ^9^ Robert‐Bosch‐Krankenhaus Stuttgart Stuttgart Germany; ^10^ Centre Hospitalier Universitaire de Grenoble La Tronche France; ^11^ Medtronic Mounds View Minnesota USA; ^12^ Medtronic Bakken Research Center Maastricht The Netherlands; ^13^ University Hospitals Leuven Leuven Belgium

**Keywords:** bradycardia, leadless pacing, Micra, outcomes, performance over time

## Abstract

**Background:**

While prior Micra trials demonstrated a high implant success rate and favorable safety and efficacy results, changes in implant populations and safety over time is not well studied. The objective of this analysis was to report the performance of Micra in European and Middle Eastern patients and compare to the Micra Investigational Device Exemption (IDE) and Micra Post Approval Registry (PAR) studies.

**Methods:**

The prospective, single‐arm Micra Acute Performance European and Middle Eastern (MAP EMEA) registry was designed to further study the performance of Micra in patients from EMEA. The primary endpoint was to characterize acute (30‐day) major complications. Electrical performance was analyzed. The major complication rate through 12 months was compared with the IDE and PAR studies.

**Results:**

The MAP EMEA cohort (*n* = 928 patients) had an implant success rate of 99.9% and were followed for an average of 9.7 ± 6.5 months. Compared to prior studies, MAP EMEA patients were more likely to have undergone dialysis and have a condition which precluded the use of a transvenous pacemaker (*p* < .001). Within 30 days of implantation, the MAP EMEA cohort had a major complication rate of 2.59%. Mean pacing thresholds were low and stable through 12 months (0.61 ± 0.40 V at 0.24 ms at implant and 12 months). Through 12 months post‐implantation, the major complication rate for MAP EMEA was not significantly different from IDE (*p* = .56) or PAR (*p* = .79).

**Conclusion:**

Despite patient differences over time, the Micra leadless pacemaker was implanted with a high success rate and low complication rate, in‐line with prior reports.

## Graphical abstract

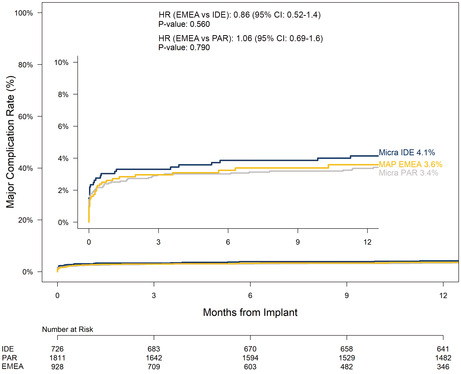

## 1 INTRODUCTION

Complications associated with traditional transvenous pacemakers (TV‐PM) frequently include pocket and lead‐related issues.^1^, ^2^ To circumvent these issues, leadless pacemakers were designed to minimize or eliminate adverse events.^3^, ^4^ The initial safety and efficacy of the Micra VR system were investigated in the Micra Investigational Device Exemption Study (IDE),^4^ wherein the device was implanted with a high success rate (99.2%) and a low complication rate at 1‐year after follow‐up (4%) compared to the TV‐PM complication rate for the historical cohort (7.6%). The Micra IDE study revealed no major infections or macro‐dislodgements with stable pacing parameters at up to 24‐month of follow‐up.

The subsequent Micra Post‐Approval Registry (PAR) was designed to investigate the safety and efficacy of Micra VR in a real‐world setting from July 2015 to January 2017.^5^ Of the 1817 patients enrolled in the Micra PAR, Micra VR was successfully implanted in 99.1% of patients with a 1‐year major complication rate of 2.7% (63% lower than patients with TV‐PM) with low and stable thresholds through 12 months. While the Micra IDE and Micra PAR studies demonstrated high implant success rates and favorable safety and efficacy results, changes in patient populations over time and the impact these changes may have on outcomes has not been well studied. To address this, the current Micra Acute Performance (MAP) study was designed to further evaluate the safety and performance of Micra when used in new non‐selected patients in the EU and Middle East. The purpose of this report is to detail the MAP regional results and compare to the previously reported Micra IDE and PAR studies.

## 2 METHODS

### 2.1 Study design

The post‐market MAP study was a prospective, non‐randomized, multicenter study conducted from February 2018 to December 2020 in Europe and the Middle East (see Table S1 for site listing) and is hereafter referred to as the MAP EMEA study. Patients enrolled in the Micra PAR were not enrolled in the MAP EMEA study and vice versa. All protocols were approved by an ethics committee at each of the participating centers, as applicable and all system‐ and procedure‐related events were adjudicated by a Clinical Events Committee of independent physicians.

### 2.2 Patients and procedures

Patients intended to be implanted with a market‐approved Micra VR device (MC1VR01) were eligible for enrollment in the study. Enrolled patients provided written informed consent.

The Micra VR is a single‐chamber ventricular pacemaker that is 93% smaller than a TV‐PM system with a total volume of 0.8 ml. The functionality and features of Micra VR are similar to existing single‐chamber ventricular pacemakers including rate adaptive pacing, remote monitoring capabilities and automated pacing capture threshold management intended to maximize battery longevity. Micra VR is intentionally designed to be magnetic resonance imaging conditionally safe for full‐body scans by both 1.5T and 3T scanners.^6^ Implantation occurred through the femoral vein into the right ventricle.^7^ Fixation of the device is in the myocardium through 4 flexible nitinol tines.^7^, ^8^

Enrolled patients underwent an implant attempt and were followed according to the provider's routine care practices. Device and patient status were reported at implant/pre‐hospital discharge, 30 days after implantation, and patients were followed approximately every 6–18 months post‐implant according to site standard of care until patient exit or study closure. All system‐ and procedure‐related adverse events including system revisions were reported immediately after center awareness.

### 2.3 End point analysis

The primary endpoint of this study was defined as major complications related to the Micra VR device or procedure that occurred within 30 days of implant. Major complications are adverse events that resulted in one or more of the following: (1) death, (2) permanent loss of device function due to mechanical or electrical dysfunction of the device, (3) hospitalization, (4) prolonged hospitalization by 48 h or more and (5) system revision (explant, repositioning, or replacement). All events were diagnosed by center investigators and appropriately reported. The Clinical Events Committee adjudicated and reviewed all related adverse events to determine whether each incidence was related and determine whether each event met the criteria for a major complication. The major complication rates at 12 months were compared with those from the Micra IDE and PAR studies. Electrical performance at implantation/prehospital discharge and follow‐up was also characterized.

### 2.4 Statistical analysis

Baseline variables were described in terms of their mean ± standard deviation, median, IQR, range, frequency counts, and/or sample proportions, as appropriate for their respective distributions and types. Baseline covariates were compared between cohorts using generalized linear models for continuous variables and Fisher's exact test for categorical variables.

For the primary analysis involving the risk of major complications, 12‐month estimates were obtained using the Kaplan–Meier method, while major complication rates were compared between cohorts using the Fine‐Gray competing risks model to account for mortality unrelated to the device or procedure. Electrical parameters (pacing capture threshold, impedance, and R‐wave amplitude) were captured both at in‐person visits and through remote transmission but were aggregated into approximate follow‐ups with data 8–92 days post‐implant corresponding to month 3, data 93–273 days post‐implant corresponding to month 6, and data from 274–457 days post‐implant corresponding to month 12. Electrical parameters were summarized by approximate visit using means and standard deviation. All hypothesis tests were two‐sided with α = 0.05. Analysis was done using SAS 9.4 (SAS Institute) except for graphics, which were created with R v3.6.3 (R Project).

## 3 RESULTS

### 3.1 Patients

The MAP EMEA cohort included 928 patients at 53 study centers in 14 countries located throughout Europe and the Middle East (Table S1 in the Online Resource). Characteristics of these subjects are listed in Table 1. Patients in this cohort were indicated for pacing primarily due to bradyarrhythmia with atrial fibrillation (*n* = 519, 55.9%), followed by atrioventricular block (*n* = 168, 18.1%), syncope (*n* = 132, 14.2%), and sinus node dysfunction (*n* = 86, 9.3%). Implant success was achieved in 927 of 928 patients (99.9%). The patient not able to be implanted was exited from the study without a device due to difficulty inserting the introducer as a result of prior catheter ablation. The mean follow‐up duration was 9.7 ± 6.5 months (0–32.7 months).

**TABLE 1:** Baseline characteristics

Subject characteristics	IDE (*N* = 726)	PAR (*N* = 1811)	MAP EMEA (*N* = 928)	*p*‐value
Age				
Mean ± standard deviation	75.9 ± 11.0	75.6 ± 13.4	76.3 ± 13.2	.48
Gender (% female)	41.2% (299/726)	38.8% (702/1809)	37.6% (349/928)	.33
Co‐morbidities				
Atrial tachyarrhythmias	75.5% (548/726)	76.0% (1374/1809)	72.2% (670/928)	.09
CHF	18.0% (131/726)	13.0% (236/1809)	8.3% (77/928)	<.001
COPD	12.7% (92/726)	9.8% (177/1809)	10.9% (101/928)	.10
CAD	28.2% (205/726)	22.0% (398/1809)	19.9% (185/928)	<.001
HTN	78.7% (571/726)	64.9% (1174/1809)	64.9% (602/928)	<.001
Diabetes	28.5% (207/726)	26.5% (480/1809)	30.2% (280/928)	.12
Renal dysfunction	20.5% (149/726)	21.5% (389/1809)	28.9% (268/928)	<.001
Dialysis	3.9% (28/726)	7.9% (143/1809)	10.2% (95/928)	<.001
Prior CIED	N/A	13.2% (239/1811)	20.4% (189/928)	<.001
Condition that precludes the use of TV‐PPM	6.2% (45/726)	23.9% (433/1808)	44.0% (408/928)	<.001
Venous access issues (including thrombosis)^a^	4.7% (34)	5.6% (101)	9.1% (84)	
History of CIED infection/bacteremia	0.6% (4)	9.4% (171)	22.3% (207)	
Cancer	0.8% (6)	2.2% (39)	5.9% (55)	
Other reason	0.8% (6)	7.6% (137)	7.9% (73)^b^	
Pacing indication (%)				
Bradyarrhythmia with AF	63.9% (464/726)	62.6% (1128/1803)	55.9% (519/928)	<.001
Sinus node dysfunction	17.4% (126/726)	9.7% (174/1803)	9.3% (86/928)	
AV block	15.0% (109/726)	11.6% (210/1803)	18.1% (168/928)	
Syncope	2.2% (16/726)	13.5% (243/1803)	14.2% (132/928)	
Other	1.5% (11/726)	2.7% (48/1803)	2.5% (23/928)	
Not reported	0.0% (0/726)	0.0% (0/1803)	0.0% (0/928)	
Follow‐up (months)				
Mean ± standard deviation	31.8 ± 23.4	31.3 ± 15.5	9.7 ± 6.5	N/A
Median	19.6	34.2	9.6	
25th Percentile–75th Percentile	14.5–55.3	21.9–42.9	3.8–14.3	
Minimum–Maximum	0.0–82.1	0.0–62.4	0.0–32.7	
Number of subjects with measure available (*N*, %)	726 (100.0%)	1811 (100.0%)	928 (100.0%)	

Abbreviations: AF, atrial fibrillation; AV, atrioventricular; CAD, chronic arterial disease; CHF, chronic heart failure; CIED, cardiac implantable electronic device; COPD, chronic obstructive pulmonary disease; HTN, hypertension; N/A, not applicable; TV‐PPM, transvenous permanent pacemaker.

^a^ Venous access issues include venous anatomy, occlusion, or need to preserve veins for hemodialysis.

^b^ Other reasons include 47 patients with valve issues, 17 patients with a prior complication to a prior CIED, and 9 miscellaneous/unknown.

Compared to the IDE and PAR cohort, MAP EMEA patients were less likely to have heart failure (8.3% vs. 18.0% and 13.0%; *p* < .001) and coronary artery disease (19.9% vs. 28.2% and 22.0%; *p* < .001) and more likely to have renal dysfunction (28.9% vs. 20.5% and 21.5%; *p* < .001) and be on dialysis (10.2% vs 3.9% and 7.9%; *p* < .001). MAP EMEA patients were also more likely to have a prior CIED implant. More patients in the MAP EMEA cohort had a condition which precluded the use of TV‐PPM (44.0% vs. 6.2% and 23.9%; *p* < .001).

### 3.2 Implant characteristics

Of the patients implanted with a Micra device in the MAP EMEA cohort, 721 were implanted by an electrophysiologist (77.7%) and 207 were implanted by an interventional cardiologist. Compared to Micra IDE and Micra PAR, the MAP EMEA cohort had a significantly shorter procedure duration (25.0 ± 26.0 min vs. 34.8 ± 24.0 min and 32.8 ± 25.6 min; *p* < .001) (Table S2 in the Online Resource). The MAP EMEA cohort also had a lower fluoroscopy duration compared to Micra IDE and Micra PAR (6.4 ± 5.9 min vs. 8.9 ± 16.6 min and 9.5 ± 17.4 min; *p* < .001). Additionally, 90.4% of the MAP EMEA cohort required ≤3 deployments.

### 3.3 Safety

Within 30 days of implantation, 24 major complications were reported in 24 patients (2.59% complication rate; 95% CI: 1.66%–3.82%) (Table 2). Of the 24 acute major complications, there were 10 events at the groin and puncture site, 6 cardiac effusion/perforation events, 4 device pacing issues, 3 infection events (2 resulting in system revisions), and 1 other event (hemodynamic instability). Through study follow‐up, there were an additional 11 major complications occurring after 30 days in an additional 9 patients adjudicated as related to the Micra VR device or procedure (see Table 2 for details).

**TABLE 2:** Major complication listing for MAP EMEA

No. events (no. subjects, %)	MAP EMEA (*n* = 928)	
Adverse event	Acute (≤30 days)	Total
Total major complications	24 (24, 2.59%)	35 (33, 3.56%)
Events at groin puncture site	10 (10, 1.08%)	10 (10, 1.08%)
Arterial injury/arteriovenous fistula fistula	4 (4, 0.43%)	4 (4, 0.43%)
Hematoma	4 (4, 0.43%)	4 (4, 0.43%)
Incision site hemorrhage	2 (2, 0.22%)	2 (2, 0.22%)
Cardiac effusion/perforation	6 (6, 0.65%)	7 (7, 0.75%)
Cardiac tamponade	3 (3, 0.32%)	3 (3, 0.32%)
Pericardial effusion	3 (3, 0.32%)	3 (3, 0.32%)
Pericardial haemorrhage	0 (0, 0%)	1 (1, 0.11%)
Pacing issues	4 (4, 0.43%)	7 (5, 0.54%)
Device capturing issue	1 (1, 0.11%)	1 (1, 0.11%)
Device pacing issue	3 (3, 0.32%)	5 (4, 0.43%)
Device stimulation issue	0 (0, 0%)	1 (1, 0.11%)
Cardiac rhythm disorder	0 (0, 0%)	1 (1, 0.11%)
Ventricular dyssynchrony	0 (0, 0%)	1 (1, 0.11%)
Infection	3 (3, 0.32%)	4 (4, 0.43%)
Bacteraemia	1 (1, 0.11%)	1 (1, 0.11%)
Endocarditis	0 (0, 0%)	1 (1, 0.11%)
Pulmonary sepsis	1 (1, 0.11%)	1 (1, 0.11%)
sepsis	1 (1, 0.11%)	1 (1, 0.11%)
Other	1 (1, 0.11%)	6 (6, 0.65%)
Cardiac failure	0 (0, 0%)	2 (2, 0.22%)
Haemodynamic instability	1 (1, 0.11%)	1 (1, 0.11%)
Pacemaker syndrome	0 (0, 0%)	2 (2, 0.22%)
Tricuspid valve incompetence	0 (0, 0%)	1 (1, 0.11%)

### 3.4 System revisions

Of the major complications, 11 led to system revisions in 10 patients. Reasons for revisions included 3 elevated pacing thresholds (in 2 patients), 3 device upgrades (1 CRT‐D, 1 CRT‐pacemaker, 1 transvenous single‐chamber pacemaker for His‐bundle pacing), 2 infections, 2 pacemaker syndrome events (both upgraded to a dual chamber transvenous pacing system), and 1 programming of the device to OOO due to lack of improvement to the patient's condition. Types of system revision included 7 instances of programming Micra device off (OOO mode) in 6 patients, 2 percutaneous retrievals, 1 surgical extraction during prior lead fragment removal, and 1 programming to backup.

Of the two system revisions due to infection, one patient was diagnosed with endocarditis and sepsis 110 days post Micra implant. The patient's device was removed prophylactically 126 days post‐implant during a surgery to replace the mitral valve and remove vegetation on the tricuspid valve. Nine days later, the patient was implanted with a transvenous dual‐chamber pacing system after which the patient died of respiratory failure. The second patient received a sepsis diagnosis 10 days following Micra implantation, with a transesophageal echocardiogram showing suspected vegetation on their tricuspid valve and a leftover fragment from an old pacing lead. The patient had their lead fragment and Micra device surgically extracted 43 days post‐implant and replaced with an epicardial pacing system with concomitant valvuloplasty.

### 3.5 Perforations/effusions

There were a total of 9 perfusion events in 9 patients (0.97%). Of these events, 7 met the criteria for a major complication (0.75%; 95% CI: 0.30–1.55%). None required surgical intervention, but 5 patients were treated with pericardiocentesis/pericardial drainage. One patient had a hemodynamically, marginally relevant, pericardial effusion and underwent pericardiocentesis. During the preparation of the pericardial puncture the patient went pulseless, and following resuscitation and pericardial puncture, spontaneous circulation returned. After the pericardial drain was placed and completion of sedatives, the patient experienced epileptic seizures. The pericardial drain was removed and the patient's condition worsened despite drug therapy and treatment. The patient died of multiple organ failure 11 days post‐implantation with cerebral spasm and septic shock arising from a urinary tract infection. The site deemed that the pericardial effusion was due to the Micra‐implant procedure but was unable to determine if the effusion was related to the Micra introducer or device.

A recent risk score analysis identified BMI ≤20, age ≥85 years, being female, no pacing indication for AF, and chronic obstructive pulmonary disease as risk factors for pericardial effusion with Micra.^9^ Based upon risk score categories, there were 5 perforations in elevated (medium/high) risk patients (out of 248 elevated risk patients; 2.0%) and 4 perforations in low risk patients (out of 647 low risk patients; 0.6%). For the 9 patients with perforations, the associated risk factors and outcomes are detailed in Table S3.

### 3.6 Deaths

There were 127 deaths reported during the study, of which 2 (0.22%; 95% CI: 0.03%–0.78%) were considered related to the Micra system or procedure. One death occurred following a pericardial effusion as detailed above. The second death occurred in a patient who was hospitalized for 9 days for the extraction of pacing leads and angioplasty of the superior vena cava. During the same procedure and following extraction, the patient underwent Micra implantation. However, immediately following the completion of implant, the patient became noradrenaline‐dependent, and their condition worsened with hemodynamic instability. Sepsis was suspected and broad‐spectrum antibiotic treatment was administered. However, the patient's renal function rapidly degraded and was followed by respiratory distress, chest pain, and hyperlactatemia with no known etiology. The patient subsequently went into cardiac arrest and died 3 days post‐implant.

### 3.7 Electrical performance

**FIGURE 1:**
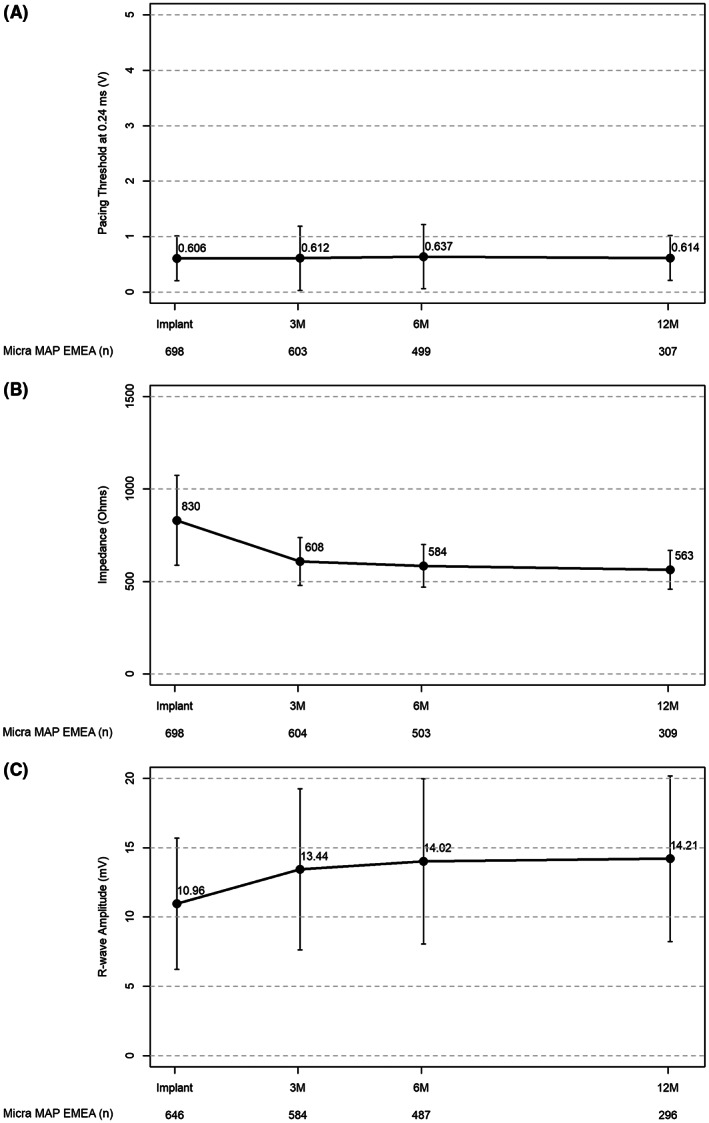
Micra electrical parameters by timepoint. Pacing threshold (A), impedance (B), and R‐wave amplitude (C) were collected for MAP EMEA patients over 12 months

The mean pacing capture threshold was 0.61 ± 0.40 V at 0.24 ms (*n* = 698) at implantation and remained stable through 12 months of follow‐up (0.61 ± 0.40 V at 0.24 ms) (*n* = 307) (Figure 1). Of the 307 patients with available pacing threshold data at 12 months, 98.1% had a pacing threshold of ≤2 V (*n* = 301). The mean impedance was 830.3 ± 243.1 Ω at implantation (*n* = 698) and 563.2 ± 104.9 Ω at 12 months (*n* = 309). The mean R‐wave amplitude was 11.0 ± 4.7 mV at implantation and 14.2 ± 6.0 mV (*n* = 646) at 12 months (*n* = 296).

### 3.8 Safety: MAP EMEA cohort vs IDE and PAR

**FIGURE 2:**
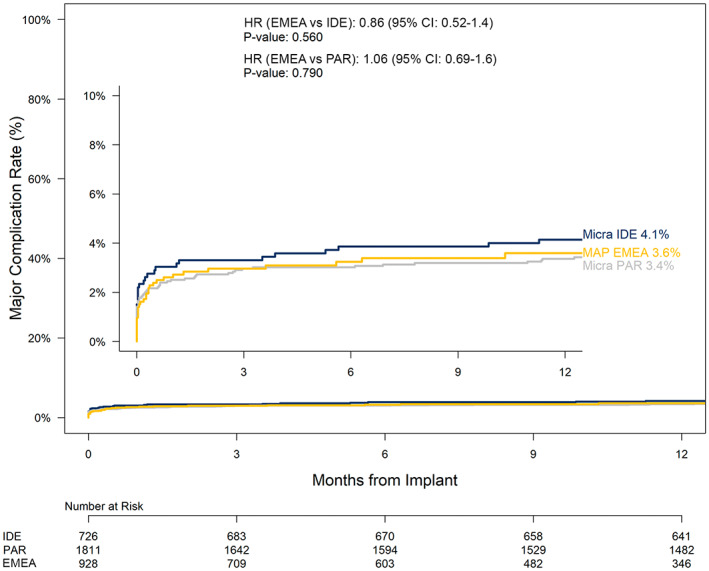
Major complication rates through 12 months for MAP EMEA, Micra IDE, and Micra PAR study cohorts. Kaplan–Meier analysis of major complications over 12 months. Fine‐gray competing risks were used to compare major complication rates. EMEA, Europe, Middle East, and Africa; IDE, investigational device exemption; PAR, post‐approval registry

At 30 days post‐implant, there was no significant difference in major complication rates between MAP EMEA and Micra IDE (*p* = .465) and MAP EMEA and Micra PAR (*p* = .564). Through 12 months post‐implantation, the major complication rate in the MAP EMEA cohort was 3.6% compared to 4.1% for Micra IDE and 3.4% for Micra PAR (Figure 2). The risk for major complication through 12‐month was not significantly different between the MAP EMEA cohort and Micra IDE (HR: 0.86; 95% CI: 0.52–1.40, *p* = .56). Similarly, there was no significant difference in risk for major complication through 12‐month between the MAP EMEA cohort and Micra PAR (HR: 1.06; 95% CI: 0.69–1.60, *p* = .79).

## 4 DISCUSSION

The first Micra VR device was implanted in 2013 and subsequently, has been studied in detail through the IDE study and PAR. These studies have shown that Micra can be implanted with high success rates and with complication rates comparable, if not better, than conventional transvenous pacing systems.^4^, ^10^, ^11^ The MAP EMEA study provides further evidence that these outcomes continue with ongoing implantation in new centers and new geographies. This study has shown an implant success rate of 99.9% (vs. 99.2% and 99.1% for the IDE and PAR, respectively) and an acute major complication rate of 2.59% (vs. 3.03% and 2.48% for the IDE and PAR, respectively). It is important to study outcomes of new technologies above and beyond initial regulatory studies/registries to ensure ongoing efficacy and safety. These data most likely reflect the novel design of the technology and the training process in place for new implanters. This study has also demonstrated important differences in the patient cohort compared to the initial studies.

The MAP EMEA study had less patients with CHF compared to both the IDE study and PAR. It is not clear why this difference exists. It is possible that it reflects the limitations of RV only pacing in a matured technology whereas in earlier experience the benefits of the leadless aspect of pacing may have been considered more important.

There was a higher proportion of patients with renal dysfunction and on hemodialysis in this study compared to both prior studies. It is recognized that transvenous pacing in both of these groups has more complications compared to a population without renal dysfunction/hemodialysis.^12^, ^13^, ^14^, ^15^ This high rate is most likely a consequence of the requirement for frequent vascular access for renal support, including dialysis lines and fistulae, resulting in compromised vasculature and increased rates of infection. Micra has been studied in a hemodialysis population demonstrating comparable results to patients not on dialysis.^16^ The overall rate of complication was 4.40% vs. 2.64% in the non‐dialysis population. Perhaps, more importantly, this study demonstrated no infections in the population of 201 dialysis patients compared to an 8% infection rate seen in patients implanted with TV‐PPM.^13^ This study also demonstrated equivalent results of pacing efficacy. These favorable outcomes may have influenced implanters into considering this population as particularly appropriate for Micra.

Micra has been shown to be an effective pacing modality in patients that have had a previous device related infection with extraordinarily low levels of subsequent infection. El‐Chami et al reported a series of 105 patients that received a Micra device following the extraction of a prior infected transvenous pacemaker.^17^ At follow up none of these patients demonstrated subsequent infection related to the Micra device. Within this patient population, 37% of this group had Micra implantation on the same day/sitting as the extraction procedure. This represents an opportunity to discharge patients earlier than with conventional treatment pathways where prolonged hospital stays with intravenous antibiotics are frequently followed.

Micra Acute Performance European and Middle Eastern has shown that Micra was more frequently used when conditions existed that precluded the use of a TV‐PM when compared to the prior studies (44.0% vs. 6.2% and 23.9% for MAP EMEA, IDE and PAR respectively; *p* < .001). This included issues such as venous access, previous device related infection and cancer. It is possible that this reflects on the decision making of clinicians, considering that Micra represents an elegant clinical option for this patient population. Furthermore, it is possible that reimbursement protocols mean that in some geographies Micra is not implanted in routine clinical care for uncomplicated patients that would be considered to be a traditional VVIR TV‐PPM population.

This study has highlighted significant changes in the implant procedure with shorter procedure times, less fluoroscopy, and fewer deployments than the previous studies. This may reflect the natural history of the evolution of new technologies and procedures with experience gained from early cases. It has also been recognized that there is a learning curve associated with this procedure and procedure times decrease significantly with case number.^18^, ^19^

An important finding of the study is that major complications for implantation of the Micra device remain extremely low and comparable to prior experience. As new technologies become more accepted in clinical practice the procedures are undertaken by a broader range of clinicians with varying clinical experience and volume of cases. In these circumstances a rise in complication rates might be expected. This was not seen in this study, with a 30‐day major complication rate of 2.59% (vs. 3.03% and 2.48% for IDE and PAR, respectively).

While the rate of pericardial effusion remains low (0.97%), it is important not to overlook the potential risk for this complication particularly among high‐risk patients. A recently published risk scoring algorithm for predicting risk for pericardial effusion using pre‐procedural clinical characteristics in patients undergoing Micra implant performed well when externally validated using the MAP EMEA data with a *C‐*index of 0.68 (95% CI: 0.52–0.83) despite the small number of pericardial effusion events observed.^9^ Additionally, the pericardial effusion risk increased with baseline risk level and the pericardial effusion rate increased significantly with additional Micra deployments in medium‐ and high‐risk patients. Use of this risk scoring system may help further improve patient outcomes following Micra implant. Mitigation of the risk of pericardial effusion through attention to procedural technique training (e.g., access to echocardiography equipment and pericardiocentesis kits; recognize the clinical signs and symptoms of pericardial effusion; use of contrast and advanced imaging to confirm septal placement and reduce the need for recapture) is necessary particularly among high‐risk patients.

This study builds upon the previously established safety and efficacy by extending the findings for Micra across a broader range of centers and patient profiles. In particular, the MAP EMEA cohort has a large proportion of precluded patients who have previously been reported to have a higher comorbidity profile than non‐precluded patients.^20^ Despite this difference, the safety profile in the MAP EMEA cohort is similar to previous reported findings.

### 4.1 Limitations

The MAP EMEA study has several limitations. The study was not a randomized controlled study, with the performance of the device in the MAP EMEA study being compared to prior Micra studies. The patient cohort is limited to European and Middle Eastern patients only and therefore the results may not be translatable to other patient populations. Additionally, there was a limited follow‐up duration for this study; however, despite the limited follow‐up, it is reassuring that the results are still in line and consistent with previously published studies.

## 5 CONCLUSIONS

The MAP EMEA study has shown that implantation of the Micra leadless pacemaker continues to remain a highly effective and safe procedure. This remains the case in both a traditional cohort of patients being considered for TV‐PPM and more specialized groups, such as those with renal impairment/hemodialysis, prior CIED infection or where conditions exist that preclude the use of TV‐PPM. As this procedure and device continues to evolve it will be important to evaluate its ongoing efficacy and safety.

## FUNDING INFORMATION

The Micra Acute Performance study was funded by Medtronic, Inc.

## CONFLICT OF INTEREST

Dr. Roberts reports having received honoraria from Medtronic, Inc and Boston Scientific. Dr. Clémenty reports having received consulting fees from Medtronic, Inc; Dr. Winter reports having received lecture fees from ZOLL‐CMS Germany, Medtronic, Inc. and Biotronik. Mr. Anderson, Mrs. Pol and Dr. Butler report being employees and shareholders of Medtronic, Inc. Dr. Garweg reports receiving consultant fees from Medtronic, Inc. The remaining authors have nothing to disclose.

## ETHICAL APPROVAL

This study was performed in accordance with the ethical standards set forth by the Declaration of Helsinki, and the study protocol was approved by an ethics committee from each participating institution.

## INFORMED CONSENT

Written informed consent was obtained from all participants included in the study.

## CLINICAL TRIALS REGISTRATION NUMBER

NCT02536118.

## Supporting information


Table S1–S3

## Data Availability

Data cannot be shared for ethical/privacy reasons.
