# Supplementary material for: A leadless pacemaker in the real‐world setting: Patient profile and performance over time
Source: J Arrhythm. 2023 Jan 8;39(1):1–9. doi: 10.1002/joa3.12811 (PMC9885317; doi:10.1002/joa3.12811)
Supplement: Supplementary file 1 — Table S1–S3 [file JOA3-39-1-s001.docx]

**Supplementary Table 1. Study enrollment by site**

| **Country** | **Center** | **Site Principal Investigator** |
| --- | --- | --- |
| BELGIUM | Cliniques Universitaires Saint-Luc | Dr. Marchandise  (previous PI: Prof. Le Polain de Waroux) |
|  | UZ Leuven | Prof. C. Garweg |
| CZECHIA | Na Homolce Hospital | Prof. P. Neuzil |
|  | Gentofte Hospital | Dr. T.B. Lindhardt |
| DENMARK | Odense University Hospital | Dr. J.B. Johansen |
|  | CHRU Tours - Hôpital Trousseau | Dr. N. Clémenty |
|  | CHRU de Lille | Prof. D. Klug |
|  | CHU Toulouse - Hôpital Rangueil | Dr. P. Mondoly |
|  | CHUR Brest | Prof. J. Mansourati |
|  | Capio - Clinique du Tonkin | Dr. H. Poty |
|  | Cen Hosp Univ Saint Étienne - Hôpital Nord | Prof. A. Da Costa |
| FRANCE | Centre Hospitalier Universitaire de Clermont-Ferrand | Prof. R. Eschalier |
|  | Centre Hospitalier Universitaire de Grenoble | Prof. P. Defaye |
|  | Clinique Pasteur | Dr. S. Boveda |
|  | Hôpital Arnaud de Villeneuve | Prof. J.L. Pasquié |
|  | Hôpital Haut-Lévêque - CHU de Bordeaux | Prof. P. Bordachar  (previous PI: Dr. P. Ritter) |
|  | Hôpital Pontchaillou - CHU de Rennes | Prof. C. Leclercq |
|  | Eberhard Karls Universität Tübingen Universitätsklinikum | PD Dr. med. K. Müller  (former PI: Dr. J. Schreieck) |
|  | Herzzentrum Dresden GmbH Universitätsklinik | Dr. T. Gaspar  (former PI: Dr. C. Piorkowski) |
|  | Klinikum der Universität Regensburg | Prof. Dr. L. Maier |
|  | Medizinische Hochschule Hannover | Prof. Dr. C. Veltmann |
|  | Robert-Bosch-Krankenhaus | Dr. C. Theis |
|  | SLK-Kliniken Heilbronn GmbH | Prof. Dr. med. M. Hennersdorf |
| GERMANY | Schwarzwald-Baar Klinikum Villingen-Schwenningen | Prof. Dr. W. Jung |
|  | St. Vinzenz Hospital Köln | Dr. S. Winter |
|  | Städtische Kliniken München GmbH - Klinikum Bogenhausen | Dr. med. S. Reif |
|  | Städtisches Klinikum Karlsruhe GmbH | Dr. M. Merkel |
|  | Uniklinikum Heidelberg | Prof. Dr. Z. Kaya |
|  | Universitaets-Herzzentrum Freiburg Bad Krotzingen | Dr. C. Restle |
|  | Universitatsklinikum Essen | Prof. Dr. T Rassaf |
| HUNGARY | Semmelweis University | Prof. Dr. B. Merkely |
| ICELAND | Landspitali - National hospital of Iceland | Dr. S. Gizurarson |
|  | Hadassah Medical Organization | Dr. D. Luria |
| ISRAEL | Sheba Medical Center Tel Hashomer | Dr. R. Beinart |
|  | The Barzilai Medical Center Ashkelon | Dr. V. Khalameizer |
| NETHERLANDS | UMC St Radboud | Dr. S. Westra |
|  | Akershus Universitetssykehus | Dr. A. Strand |
| NORWAY | Haukeland Universitetssjukehus | Dr. H. Keilegavlen |
|  | St. Olavs Hospital – Universitetssykehuset Trondheim | Dr. O.C. Mjølstad |
| SAUDI ARABIA | Prince Salman Heart Centre-King Fahad | Dr. F.l M. Al Smadi |
| SERBIA | Clinical Center of Serbia | Prof. S. Pavlović |
|  | Inselspital - Universitätsspital Bern | Prof. Dr. med. H. Tanner |
| SWITZERLAND | UniversitatsSpital Zurich | Dr. med. A. Breitenstin  (previous PI: Prof. Dr. Jan Steffel) |
|  | Universitätsspital Basel | Prof. Dr. med. C. Sticherling |
|  | Bristol Heart Institute | Dr. E.R. Duncan |
|  | Central Manchester University Hospitals NHS | Dr. Amir Zaidi |
| UNITED KINGDOM | Northampton General Hospital | Dr. D. Sharman |
|  | Nottingham University Hospitals NHS Trust City Hosp | Dr. T. Robinson |
|  | Queen Elizabeth Hospital | Dr. F. Leyva |
|  | Southampton General hospital | Dr. P. Roberts |
|  | St. Bartholomew's Hospital | Dr. S. Sporton |
| UNITED KINGDOM | The James Cook University Hospital-South Tees Hospitals NHS | Prof. N Linker |
|  | The Leeds Teaching Hospitals NHS Trust | Dr. C.B. Pepper |

**Supplementary Table 2. Procedural characteristics**

| **Subject Characteristics** | **IDE (N = 726)** | **PAR (N = 1811)** | **MAP EMEA (N = 928)** | **p-value** |
| --- | --- | --- | --- | --- |
| **Implant Success** | 99.2% (720/726) | 99.1% (1794/1811) | 99.9% (927/928) | 0.024 |
| **≤3 Deployments (%)** | N/A | 1522 (84.0%) | 839 (90.4%) |  |
| **Procedure Duration (min)** |  |  |  |  |
| Mean ± Standard Deviation | 34.8 ± 24.0 | 32.8 ± 25.6 | 24.2 ± 15.4 | <0.001 |
| Median | 28.0 | 26.0 | 20.0 |  |
| 25^th^ Percentile - 75^th^ Percentile | 21.0 - 41.0 | 19.0 - 40.0 | 15.0 - 29.0 |  |
| Minimum - Maximum | 7.0 - 280.0 | 1.0 - 549.0 | 2.0 - 147.0 |  |
| Number of Subjects With Measure Available (N,%) | 720 (99.2%) | 1607 (88.7%) | 815 (87.8%) |  |
| **Fluoroscopy Duration (min)** |  |  |  |  |
| Mean ± Standard Deviation | 8.9 ± 16.6 | 9.5 ± 17.4 | 6.4 ± 5.9 | <0.001 |
| Median | 6.0 | 6.6 | 5.1 |  |
| 25^th^ Percentile - 75^th^ Percentile | 4.0 - 10.0 | 4.2 - 10.9 | 3.2 - 7.8 |  |
| Minimum - Maximum | 1.0 - 387.0 | 0.0 - 490.2 | 0.3 - 100.1 |  |
| Number of Subjects With Measure Available (N,%) | 714 (98.3%) | 1723 (95.1%) | 898 (96.8%) |  |

**Supplementary Table 3: Patients with Pericardial Effusion**

| **Patient** | **Day Diagnosed^1^** | **Outcome/**  **Actions** | **Deployments** | **Age** | **BMI** | **Sex** | **CHF** | **CAD** | **MI** | **PH** | **COPD** | **AF** | **Prior Cardiac Surgery** | **Dialysis** | **Risk Score^2^** |
| --- | --- | --- | --- | --- | --- | --- | --- | --- | --- | --- | --- | --- | --- | --- | --- |
| 1 | 0 | Death | 2 | 89 | 24.3 | Male | + | + | - | + | - | - | + | - | 1 |
| 2 | 0 | Pericardiocentesis | 0 | 62 | 23.2 | Female | - | - | - | - | - | - | - | - | 1 |
| 3 | 0 | Pericardiocentesis | 1 | 89 | 25.3 | Female | + | - | - | - | - | + | + | - | 1 |
| 4 | 0 | Pericardiocentesis | 1 | 74 | 29.8 | Male | - | + | - | + | - | + | + | - | -2 |
| 5 | 0 | Pericardiocentesis | 1 | 72 | 32.2 | Male | - | - | - | - | - | - | + | + | 0 |
| 6 | 31 | Pericardiocentesis | >5 | 72 | 29.4 | Male | - | + | + | - | + | + | - | + | 2 |
| 7 | 1 | Observation | 1 | 81 | 21.6 | Female | - | - | - | + | - | - | - | - | 1 |
| 8 | 0 | Observation | 2 | 69 | 24.2 | Male | - | - | - | - | - | - | - | - | 0 |
| 9 | 1 | Observation | >5 | 72 | 30.5 | Female | - | + | - | - | - | + | - | + | 0 |

^1^Days relative to implant procedure (day 0 is day of implant).

^2^Risk score for pericardial effusion^9^. Risk scores of ≤0 indicate low risk, 1 = medium risk = 1, and ≥2 indicate high risk.

NR=not reported, ND=no deployments, CHF=congestive heart failure, CAD=coronary artery disease, COPD=chronic obstructive pulmonary disorder, MI=myocardial infarction, PH=pulmonary hypertension, AF=atrial fibrillation. Observation means the event resolved without the need for surgical intervention or pericardial drain.
